# Push-out bond strength of different endodontic obturation material at three different sites - *In-vitro* study

**DOI:** 10.4317/jced.53647

**Published:** 2017-06-01

**Authors:** Pankaj Mishra, Anjna Sharma, Sunil Mishra, Manas Gupta

**Affiliations:** 1 MDS, Senior Lecturer , Department of Conservative Dentistry and Endodontics, Rishiraj College of Dental Sciences and Research Centre, Bhopal, Madhya Pradesh, India; 2 MDS, Post Graduate student , Department of Conservative Dentistry and Endodontics, Rishiraj College of Dental Sciences and Research Centre, Bhopal, Madhya Pradesh, India; 3 MDS, Reader, Department of Maxillofacial Prosthodontics and Implantology, Peoples College of Dental Sciences & Research Centre, Bhopal, Madhya Pradesh, India; 4 MDS, Senior Lecturer , Department of Oral Medicine and Radiology, Rishiraj College of Dental Sciences and Research Centre, Bhopal, Madhya Pradesh, India

## Abstract

**Background:**

The key to success of any root canal therapy is adequate obturation of the prepared root canal space. Root canal sealers are not dimensionally stable and might dissolve partially over a period of time. The objective of this *in vitro* study is to evaluate the push-out bond strength to intraradicular dentin of two endodontic obturation materials.

**Material and Methods:**

Forty extracted single rooted permanent teeth were used. Canals orifice was explored, teeth were instrumented. The samples were divided into two groups each containing twenty specimens obturated with different obturation material (Group1 Epiphany/Resilon and Group 2 Gutta Percha/AH Plus).The obturation systems used in this study was Element Obturation unit (Sybron Endo). Each tooth root was horizontally sectioned in approximately 2-mm thick slices from the coronal 1/3rd, middle 1/3rd and apical 1/3rd. The push-out bond strength of each specimen was calculated using Universal Testing Machine. The statistical analysis was done using two way analysis of variance (ANOVA) and tukey’s test.

**Results:**

There was significant difference between push out bond strength of Resilon/Epiphany and AH Plus/Gutta Percha. Gutta percha group was superior with push out bond strength of 2.22 (± 0.16) Mpa in comparison to Resilon/Epiphany group with 1.61 (±0.14) Mpa (*p*<0.001).

**Conclusions:**

The interfacial bond strength achieved with Resilon/Epiphany self-etch (SE) to intraradicular dentine was not superior to that of AH Plus/Gutta Percha.

** Key words:**AH Plus, Apical leakage, Epiphany, Gutta percha, Push-out test Resilon.

## Introduction

One of the keys to successful root canal therapy is to adequately obturate the prepared root canal space. Root canal obturation aims to provide a complete ﬁlling of the canal in all dimensions to create a ﬂuid-tight seal to prevent ingress of bacteria and their toxins and their ﬂow into the periapical tissues (1,2). Obturation of the canal system has historically been achieved with gutta-percha and a sealer. Most root canal ﬁllings do not completely ﬁll the root canal system (1,3). Gutta-percha does not bond to the internal tooth structure, resulting in the absence of a complete seal (4,5). To reduce apical and coronal leakage of root filled teeth, both total etch and self-etch adhesives have been employed experimentally for sealing intraradicular dentin before the obturation of root canals with gutta-percha. Root canal sealers are not dimensionally stable and might dissolve partially over time as a result of their low resistance to leakage. The use of resin cements alone for root canal obturation also creates difficulties in their removal during retreatment. These obstacles have apparently been resolved by the recent launching and intensive promotion of a polyester based thermoplastic root filling material that is claimed to be bondable to methacrylate based resins (6-9).

Resilon is thermoplastic because of the incorporation of polycaprolactone, biodegradable aliphatic polyester that has a low glass transition temperature of 62°C. It is bondable to methacrylate based resins as it contains dimethacrylate resins. The adjunctive use of self-etch adhesives and methacrylate based resin sealers with Resilon purportedly creates a monobloc between the intraradicular dentin and the root-filling material that is more resistant to both bacterial leakage and root fracture when compared with similar teeth that were root filled with gutta percha and conventional sealers (10-15). Studies have shown that a true monobloc does not exist along the entire canal, but well defined coronal barrier and bonding with much of the canal have been noted (16). Different studies were done to identify the push out bond strength of different obturating materials, sealers with interfacial dentin so as to measure their effectiveness as an endodontic obturating material (17-19) with mixed results. The objective of this *in-vitro* study is to evaluate the push-out bond strength to intraradicular dentin of two endodontic obturation materials at three different sites.

## Material and Methods

Forty clean single rooted permanent teeth were collected. The crown portion of each tooth was removed by means of a diamond disc. The pulp chamber of all the teeth was opened using an endo cavity access kit (Dentsply Mallifer, USA) with an airotar hand piece. Canals orifice was explored and working length of each tooth was determined by a K- file #10 (Dentsply Mallifer, USA), until it reached the apical foramen, subtracting 1 mm from this measurement. The root canals were irrigated with 3% sodium hypochlorite (Hyposol, Jammu& Kashmir, India) and 17% ethylene diamine tetra acetic acid (Glyde, Dentsply Mallifer, USA). After the cleaning and shaping processes, ethylene diamine tetra acetic acid (Glyde, Dentsply Mallifer, USA) for 1 minute was used for removal of smear layer followed by sterile water as a final rinse. Paper point (Glyde, Dentsply Mallifer, USA) was used to dry the canals before obturation.

The samples were divided into two groups each containing twenty specimens obturated with different obturation material; Group1 Epiphany/Resilon (Sybron Endo, Real Seal, Self Etch) and Group 2 Gutta Percha/ AH Plus (Dentsply Mallifer, U.S.A).The tested materials were used for our study are epiphany self-etch (SE) sealer with Resilon core material and AH Plus sealer with gutta-percha core material. All the specimens were obturated with Element Obturation unit (Sybron Endo, Orange, CA, California, USA) and handled according to the manufacturer instructions.

**Figure 1:**
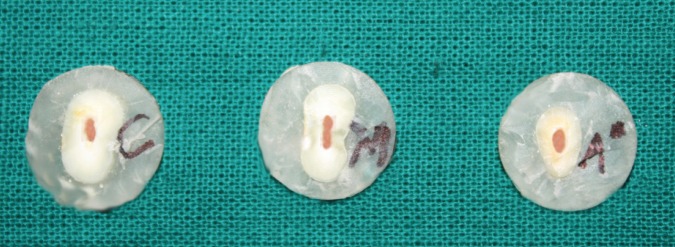
Tooth samples showing obturated tooth root.

**Figure 2:**
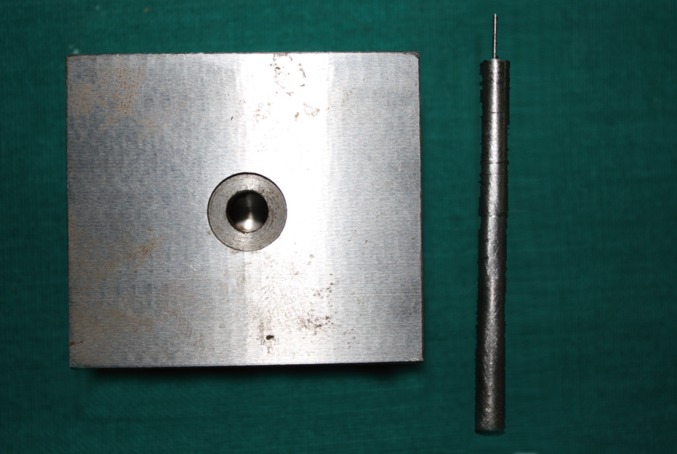
Support Jig with load cell.

**Figure 3:**
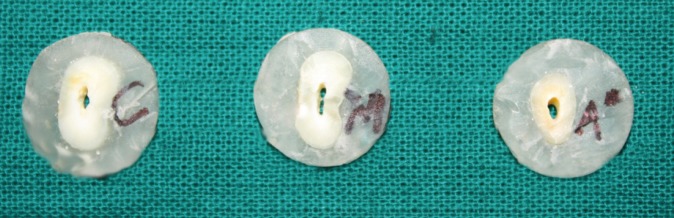
Tooth samples showing dislodged obturated material from tooth root.

After the canal obturation, the roots were radiographed to make sure the canal was fully obturated. All the specimens were embedded in resin block and were horizontally sectioned in approximately 2-mm thick slices from the coronal 1/3rd, middle 1/3rd and apical 1/3rd with a diamond disc. Each slice was marked as coronal, middle and apical to identify the sample site and side (Fig. 1). To maintain the uniformity of the sample the thickness of each slice was measured by means of a Vernier caliper. A support jig (Fig. 2) was specially fabricated for the study to mount the specimens on it while testing for the push out bond strength of the obturating material to dislodge it. Each specimen was attached to a support jig and placed on the base of the Universal Testing Machine (Instron, Norwood, MA, USA) with the coronal end of the specimen facing the support jig and the apical end facing the load cell for the punch affixed to the crosshead with a speed of 0.5 mm per minute until extrusion of the obturation (Fig. 3). The push-out bond strength value was calculated with the computer and software connected to the universal testing machine. The statistical analysis was done using two way ANOVA and tukey’s test.

## Results

The push out bond strength of two groups at three sites are summarized in  Table 1. In both groups, the mean push out bond strength was higher in apical one third followed by middle one third and least at coronalone third. The push out bond strength was higher in Gutta Percha group than Resilon group.

**Table 1:**
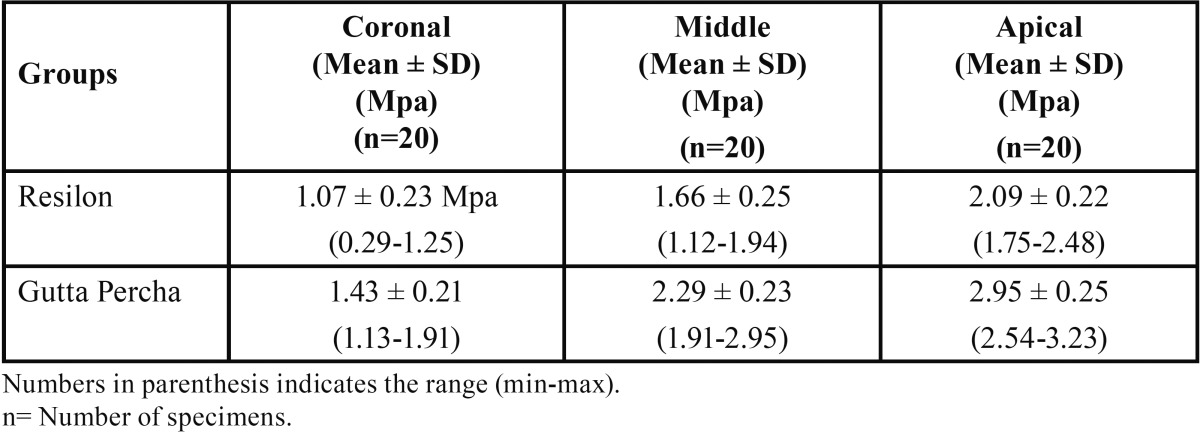
Push out bond strength of two groups at three root sites.

Comparing the mean push out bond strength of two groups and three sites ( Table 2), ANOVA revealed significantly different push out bond strength between groups (F=213.35, *p*<0.001) and among sites (F=302.15, *p*<0.001). The interaction effect of both (groups and sites) was also found to be significant (F=11.46, *p*<0.001).

**Table 2:**
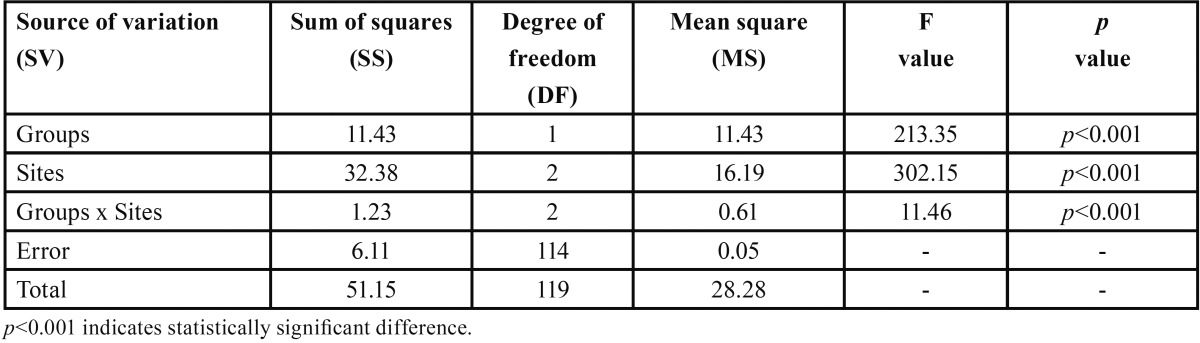
Two way ANOVA of push out bond strength between groups and root sites.

For each group, comparing the mean push out bond strength within the groups (i.e. between root sites) ( Table 3), the push out bond strength at middle and apical of Resilon group was found to be significantly (*p*<0.01and *p*<0.001 respectively) different and higher as compared to at coronal. Further, in Resilon group, the mean push out bond strength at apical was also found to be significantly (*p*<0.001) different and higher as compared to at middle.

**Table 3:**
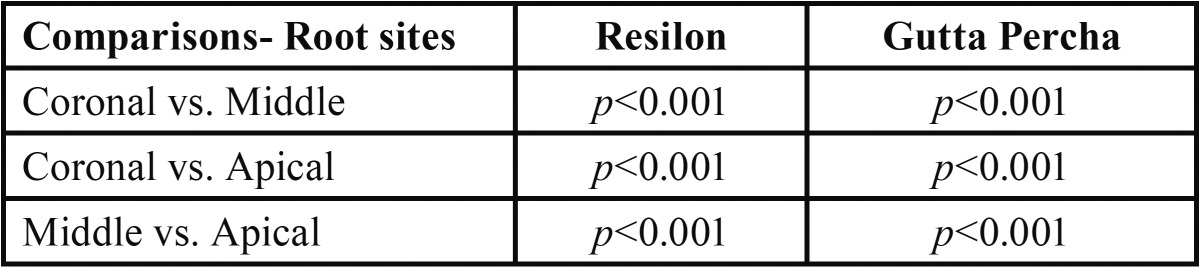
For each group, significance (*p* value) of mean difference of push out bond strength within the groups (i.e. between sites) by Tukey test.

Similarly, in Gutta Percha, the mean push out bond strength at middle and apical was also found to be significantlydifferent (p<0.001) and higher as compared to at coronal. Further, in Gutta Percha group, it was also found to be significantly different and higher at apical as compared to at middle.

Similarly, for each site, comparing the mean push out bond strength between the groups ( Table 4), the push out bond strength at Coronal, Middle and Apical of Gutta Percha group was found to be significantly (*p*<0.001) different and 25.3%, 27.6% and 29.1% higher respectively as compared to Resilon.

**Table 4:**
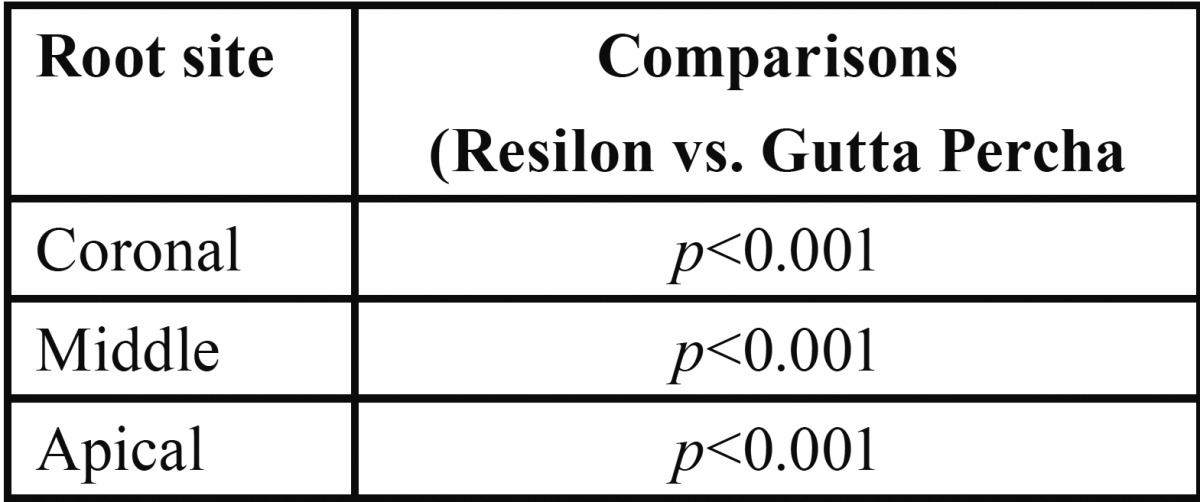
For each site, significance (*p* value) of mean difference of push out bond strength between the groups by Tukey test.

The overall push out bond strength of Resilon and Gutta Percha groups ranged from 1.30-1.83 MPa and 1.99-2.64 MPa, respectively with mean (± SD) 1.61 ± 0.14 MPa and 2.22 ± 0.16 Mpa, respectively. The overall mean push out bond strength of Gutta Percha group evident comparatively higher than Resilon group. Comparing the overall mean push out bond strength of two groups, t test revealed significantly different and 27.8% higher push bond strength of Gutta Percha group as compared to Resilon group (1.61 ± 0.14 and 2.22 ± 0.16 respectively, t=13.27; *p*<0.001).

## Discussion

The success of endodontic treatment depends on completely filling the root canal system. The goal during obturation is to fill the entire root canal system and must seal the canal both apically and coronally using materials that have physico-chemical and biological properties (18). Root canal sealers are binding agents that adapt the rigid obturating materials to the canal wall, fill up the voids, accessory canals and irregularities within the canal. Endodontic literature reports that sealer plays an important role to seal the root canal in a hermetic way. To seal a root canal with the help of sealer and obturating material means to fill it in all its extension with an inert, antiseptic material, obtaining the most hermetic seal possible. In the present study, the result showed that the interfacial push-out bond strength achieved with Resilon/Epiphany self-etch (SE) to intra- radicular dentin is not superior to that of AH Plus/Gutta Percha. The result was in accordance with the study by Marilia *et al.* (17), Ureyen *et al.* (18) and Ungor *et al.* (20), The explanation for this might be due the resin matrix material preferentially penetrated the dentinal tubules, leaving a sealer layer that is enriched with ﬁller particles that are larger than the dentinal tubule diameter. Thus a weak bond would result due to the excessively large particle ratio in the sealer layer. It has also been suggested that low concentration of dimethacrylate that is present in matrix component of Resilon might be a possible reason for absence of free radicals within polymerized Resilon material resulting in less coupling of Resilon material with the Epiphany sealer thus causes low bond strength between the Epiphany sealer and Resilon (1,10,20,21). Contrary to the present study Skidmore *et al.* (22) found in their study that mean bond strength of to root canal dentin was significantly higher in Resilon/Epiphany group as compared to the Gutta percha/ Kerr pulp canal sealer.

During biomechanical preparation, smear layer is formed. Smear layer act as reservoir for microorganisms and can also block the extension of sealer tags in to dentinal tubules, thereby decreasing micro-mechanical adhesion. Thus smear layer should be removed (4). Polymerization shrinkage of the resin sealer might also contribute to the lower bond strength value observed in Resilon group. The amount of polymerization shrinkage depends on the type, size, and content of filler particles as well as the type of matrix used. The stress associated with this shrinkage may result in separation of the resin-based sealer from the dentinal walls and consequently, the bond strength value of this interface would decrease (23). A possible explanation for the differences in push-out bond strength is that gutta percha is more compatible than Resilon, which might help resist its dislodgement (17).

In present study in both group the push out bond strength was evident higher in Apical 1/3rd followed by Middle 1/3rd and Coronal 1/3rd the least. The possible reason might be better compaction of obturating material in apical1/3rd region because of down packing resulting in better penetration of sealer in to the dentinal tubules. These leads to less polymerization shrinkage stress of resin sealer thus decreasing voids and achieve homogenous mass at the dentine-sealer and sealer-cone interface, which increases push-out bond strength. The result were in accordance with study by Mannocci *et al.* (24) were they concluded that high values of bond strength of the dentin are associated with low densities of dentinal tubules and that apical areas of root dentin have higher bond strength than middle and coronal ones. The study has limitations as it does not simulate the oral conditions and long term sealing ability of the obturating material had not been tested.

## Conclusions

Within the limits of this study, it may be concluded as following.

1) The push-out bond strength of endodontic obturating material to intraradicular dentine interface was dependent on the type of material used. The interfacial bond strength achieved with Resilon/Epiphany self-etch (SE) to intraradicular dentine was not superior to that of AH Plus/Gutta Percha. The results challenge the concept of strengthening root canals with the new root canal filling materials.

2) In both group the push out bond strength was evident higher in Apical 1/3rd followed by Middle 1/3rd and Coronal 1/3rd the least, with higher being in AH Plus/Gutta Percha than Resilon/Epiphany self-etch (SE).
